# Logistic regression analysis of factors influencing NT-proBNP expression levels in patients with unstable angina pectoris and intervention strategies

**DOI:** 10.1097/MD.0000000000047459

**Published:** 2026-04-17

**Authors:** Zheng Sun

**Affiliations:** a Department of Emergency Internal Medicine, Bozhou People’s Hospital, Bozhou City, Anhui Province, China.

**Keywords:** influencing factors, intervention strategies, N-terminal pro-B-type natriuretic peptide, unstable angina pectoris

## Abstract

This study aims to analyze the influencing factors of N-terminal pro-B-type natriuretic peptide (NT-proBNP) expression levels in patients with unstable angina pectoris (UAP) using a logistic regression model, establish a prediction model with internal validation, and propose corresponding intervention strategies. A retrospective analysis was conducted on the clinical data of 100 UAP patients admitted to our hospital from November 2021 to October 2023. Patients were randomly divided into a training set (n = 80) and a validation set (n = 20) at a ratio of 8:2. The training set was further divided into an low NT-proBNP group (n = 40) and a high NT-proBNP group (n = 40) based on serum NT-proBNP levels at admission. Differences in baseline and clinical data between the 2 groups were compared. Variables with significant differences in univariate analysis were included in the logistic regression model to identify influencing factors of NT-proBNP expression levels in UAP patients. A nomogram prediction model was constructed. The predictive value of the model was evaluated using the receiver operating characteristic curve, calibration curve, and decision curve, and corresponding intervention strategies were formulated. There were no significant differences in baseline data between the training and validation sets (*P* > .05). In the training set, age, history of myocardial infarction, left ventricular ejection fraction (LVEF), New York Heart Association (NYHA) classification, and serum creatinine showed statistically significant differences between the observation and high NT-proBNP groups (*P* < .05). The odds ratio values for age, history of myocardial infarction, LVEF, NYHA, and serum creatinine were 1.175 (95% confidence interval [CI]: 1.096–1.248), 1.244 (95% CI: 1.185–1.460), 0.085 (95% CI: 0.074–0.096), 13.183 (95% CI: 2.824–61.532), and 1.118 (95% CI: 1.015–1.183), respectively. The nomogram model was internally validated using validation set data. The area under the receiver operating characteristic curve area under the curve for the training and validation sets was 0.866 (0.789–0.944) and 0.876 (0.801–0.951). The model showed high net benefit within the threshold range, demonstrating good applicability. Conclusion Age, history of myocardial infarction, LVEF, NYHA classification, and serum creatinine level are influencing factors of NT-proBNP levels in UAP patients.

## 1. Introduction

Unstable angina pectoris (UAP) refers to angina caused by myocardial ischemic changes due to coronary artery stenosis or occlusion and is one of the most common types of coronary heart disease.^[1]^ Relevant investigations show^[2]^ that the incidence of UAP exceeds 60% among patients with coronary heart disease, with middle-aged and elderly individuals being the high-risk population. The main clinical manifestations of UAP include chest tightness and chest pain at rest, nausea, and shortness of breath. The condition progresses rapidly, is difficult to cure, and if effective treatment measures are not taken in time, may develop into acute myocardial infarction or sudden death, posing a serious threat to patients’ lives.^[3]^ Data indicate^[4]^ that more than 1 million UAP patients are admitted to hospitals annually, and even with standardized and reasonable treatment, the 1-year mortality rate remains as high as 7.6%, and the incidence of adverse events is 14.9%. Therefore, the early detection, prevention, and control of UAP have gradually become hot topics of clinical concern.

N-terminal pro-B-type natriuretic peptide (NT-proBNP) is a precursor of B-type natriuretic peptide. Clinical studies have shown that this biomarker is closely associated with coronary artery disease. Due to its high sensitivity and specificity, NT-proBNP is now widely used in the diagnosis and prognosis evaluation of diseases such as myocardial infarction and heart failure.^[5-7]^ Relevant studies have indicated^[8]^ that serum NT-proBNP expression is a risk factor for UAP. The occurrence of UAP leads to myocardial ischemia and impaired ventricular function, which in turn causes an increase in NT-proBNP levels. NT-proBNP has high predictive value for cardiovascular events. However, studies on the influencing factors of NT-proBNP levels in UAP patients are currently limited, and no unified conclusion has been reached. Some scholars believe it may be related to age and atrial fibrillation,^[9]^ while others suggest it may be associated with cardiac function.^[10]^ This study analyzes the influencing factors of NT-proBNP expression levels in UAP patients using a logistic regression model and proposes relevant intervention strategies. It is expected to provide a reference for the clinical diagnosis and prognosis prediction of UAP and guide clinical practice.

## 2. Object and methods

### 2.1. Research subjects

This study was approved by the Ethics Committee of Bozhou People’s Hospital. A retrospective analysis was conducted on the clinical data of 100 patients with UAP admitted to our hospital from November 2021 to October 2023. The patients were randomly divided into a training set (n = 80) and a validation set (n = 20) at a ratio of 8:2. Based on serum NT-proBNP levels at admission, the training set was further divided into a low NT-proBNP group (n = 40) and a high NT-proBNP group (n = 40).

Diagnostic criteria: Referring to the *Guidelines for the Diagnosis and Treatment of Non-ST-Elevation Acute Coronary Syndromes (2016*)^[11]^ for UAP:

1. Presence of precordial pain or discomfort, with symptoms occurring more frequently within the past month and more severe than before;
2. During episodes, electrocardiogram (ECG) shows ST-segment depression or elevation in contiguous leads;
3. Coronary angiography reveals >50% coronary artery stenosis.

Among these, coronary angiography is the gold standard for diagnosis; however, typical symptoms combined with ECG changes during attacks may also confirm the diagnosis.

Inclusion criteria:

1. Meeting the above diagnostic criteria;
2. Age ≥ 40 years;
3. Good compliance;
4. Complete clinical data.

Exclusion criteria:

1. History of congenital heart disease, acute myocardial infarction, or heart failure;
2. Uncontrolled diabetes mellitus or hypertension;
3. Chronic hepatitis or infectious diseases;
4. Autoimmune diseases or malignancies;
5. Presence of diseases in other major organs such as the liver or kidneys;
6. Participation in other clinical studies;
7. Refusal to participate or withdrawal during the study.

### 2.2. Methods

#### 2.2.1. General data collection

General information of all patients was recorded through retrospective analysis of medical records, mainly including sex, age, body mass index (BMI), medical history, and medication use. Medical history included diabetes, hypertension, myocardial infarction, stroke, and previous percutaneous coronary intervention (PCI). Medications included β-blockers, antiplatelet drugs, calcium channel blockers, angiotensin-converting enzyme inhibitors/angiotensin receptor blockers, and statins.

#### 2.2.2. Clinical data recording and assessment

Clinical data of all patients were obtained through retrospective analysis of medical records, mainly including ECG, blood pressure, echocardiography, New York Heart Association (NYHA) cardiac function classification, complete blood count, liver and kidney function, fasting blood glucose (FBG), and blood lipid profile.

#### 2.2.3. Grouping criteria

Based on serum NT-proBNP levels at admission and using the median value for grouping, the median serum NT-proBNP level among all patients was 608.62 pg/mL. Patients with serum NT-proBNP levels less than or equal to the median were assigned to the low NT-proBNP group, while those with levels greater than the median were assigned to the high NT-proBNP group.

### 2.3. Observation indicators

The following variables were observed and compared between the 2 groups: general data, heart rate (HR), FBG, systolic blood pressure (SBP), diastolic blood pressure (DBP), triglycerides (TG), total cholesterol (TC), low-density lipoprotein cholesterol (LDL-C), high-density lipoprotein cholesterol (HDL-C), NYHA cardiac function classification, left ventricular ejection fraction (LVEF), creatinine (Cr), and uric acid (UA) levels.

### 2.4. Statistical methods

Statistical Package for the Social Sciences (SPSS 25.0, IBM Corp., Armonk) statistical software was used for data analysis. Categorical data were expressed as n (%). When sample size ≥40 and theoretical frequency *T* ≥ 5, the chi-square test basic formula was used, and the test statistic was χ²; when sample size ≥40 but 1 ≤ *T* < 5, the chi-square test with continuity correction was used; when sample size <40 or *T* < 1, Fisher’s exact test was applied. Measurement data were first tested for normality using the Shapiro–Wilk test. Normally distributed data were expressed as (*x̄* ± *s*) and analyzed using the *t*-test; non-normally distributed data were expressed as median and interquartile range [M(P25, P75)] and analyzed using nonparametric tests. A *P*-value < .05 was considered statistically significant. Additionally, the logistic regression risk prediction model was constructed using the rms package in R 3.6.2 software, and a nomogram was plotted. The predictive value of the model was evaluated by constructing receiver operating characteristic curves, calibration curves, and decision curves.

## 3. Results

### 3.1. Comparison of general data between the training set and validation set

There were no statistically significant differences between the training set and the validation set in terms of sex, age, BMI, history of diabetes, hypertension, stroke, myocardial infarction, PCI history, medication use, HR, SBP, DBP, FBG, TC, TG, HDL-C, LDL-C, LVEF, NYHA classification, UA, and Cr levels (*P* > .05; see Table 1 for details).

**Table 1:** Comparison of general data between the training set and validation set.

Indicator	Training set (n = 80)	Validation set (n = 20)	χ²/*t*	*P*
Gender (n [%])				
Male	40 (50.00)	11 (55.00)	0.16	.689
Female	40 (50.00)	9 (45.00)		
Age (*x̄* ± *s*, yr)	69.78 ± 5.14	70.24 ± 5.26	0.356	.722
Body mass index (*x̄* ± *s*, kg/m²)	23.96 ± 2.75	24.10 ± 2.84	0.165	.869
Medical history (n [%])				
Diabetes	6 (7.50)	1 (5.00)	0.154	.695
Hypertension	9 (11.25)	2 (10.00)	0.026	.873
Stroke	4 (5.00)	1 (5.00)	0.329	.566
Myocardial infarction	13 (16.25)	3 (15.00)	0.019	.892
History of PCI	3 (3.75)	1 (5.00)	0.065	.799
Medication history (n [%])				
β-blockers	38 (47.50)	9 (45.00)	0.04	.841
Antiplatelet agents	40 (50.00)	12 (60.00)	0.641	.423
Calcium channel blockers	18 (22.50)	5 (25.00)	0.056	.812
ACEI/ARB	37 (46.25)	9 (45.00)	0.01	.92
Statins	24 (30.00)	5 (25.00)	0.194	.659
HR (*x̄* ± *s*, beats/min)	68.50 ± 10.75	69.26 ± 10.82	0.282	.778
SBP (*x̄* ± *s*, mm Hg)	128.94 ± 15.80	129.45 ± 16.22	0.128	.898
DBP (*x̄* ± *s*, mm Hg)	74.32 ± 9.45	75.27 ± 10.06	0.397	.692
FBG (*x̄* ± *s*, mmol/L)	5.85 ± 1.22	6.06 ± 1.30	0.68	.498
TC (*x̄* ± *s*, mmol/L)	4.24 ± 1.05	4.29 ± 1.16	0.187	.852
TG (*x̄* ± *s*, mmol/L)	1.64 ± 0.46	1.69 ± 0.48	0.431	.667
HDL-C (*x̄* ± *s*, mmol/L)	1.14 ± 0.28	1.10 ± 0.36	0.538	.592
LDL-C (*x̄* ± *s*, mmol/L)	2.34 ± 0.68	2.37 ± 0.65	0.178	.859
LVEF (%)	64.62 ± 8.96	65.07 ± 9.33	0.199	.842
NYHA classification (n [%])			0.225	.974
Class I	29 (36.25)	10 (50.00)		
Class II	31 (38.75)	13 (65.00)		
Class III	5 (6.25)	2 (10.00)		
Class IV	15 (18.75)	5 (25.00)		
Cr (*x̄* ± *s*, μmol/L)	80.36 ± 9.86	81.24 ± 9.96	0.356	.722
UA (*x̄* ± *s*, μmol/L)	322.35 ± 49.36	325.97 ± 52.41	0.29	.773

ACEI/ARB = angiotensin-converting enzyme inhibitors/angiotensin receptor blockers, Cr = creatinine, DBP = diastolic blood pressure, FBG = fasting blood glucose, HDL-C = high-density lipoprotein cholesterol, HR = heart rate, LDL-C = low-density lipoprotein cholesterol, LVEF = left ventricular ejection fraction, NYHA = New York Heart Association, PCI = percutaneous coronary intervention, SBP = systolic blood pressure, TC = total cholesterol, TG = triglycerides, UA = uric acid.

### 3.2. Comparison of general data between the low NT-proBNP group and high NT-proBNP group in the training set

There were no statistically significant differences between the 2 groups in terms of sex, BMI, history of diabetes, hypertension, stroke, PCI, medication use, HR, SBP, DBP, FBG, TC, TG, HDL-C, LDL-C, and UA levels (*P* > .05). However, statistically significant differences were observed in age, history of myocardial infarction, LVEF, NYHA classification, and Cr levels (*P* < .05; see Table 2 for details).

**Table 2:** Comparison of general data between the observation group and control group in the training set.

Indicator	Observation group (n = 40)	Control group (n = 40)	χ²/*t*	*P*
Gender (n [%])				
Male	18 (45.00)	22 (55.00)	0.8	.371
Female	22 (55.00)	18 (45.00)		
Age (*x̄* ± *s*, yr)	68.65 ± 4.98	71.83 ± 5.14	2.81	.006
Body mass index (*x̄* ± *s*, kg/m²)	24.30 ± 2.62	23.80 ± 2.78	0.828	.41
Medical history (n [%])				
Diabetes	2 (5.00)	4 (10.00)	0.18	.671
Hypertension	4 (10.00)	5 (12.50)	0	1
Stroke	1 (2.50)	3 (7.50)	0.263	.608
Myocardial infarction	1 (2.50)	12 (30.00)	9.185	.002
History of PCI	1 (2.50)	2 (5.00)	0	1
Medication use (n [%])				
β-blockers	18 (45.00)	20 (50.00)	0.201	.654
Antiplatelet agents	19 (47.50)	21 (52.50)	0.2	.655
Calcium channel blockers	8 (20.00)	10 (25.00)	0.287	.592
ACEI/ARB	18 (45.00)	19 (47.50)	0.05	.823
Statins	11 (27.50)	13 (32.50)	0.238	.626
HR (*x̄* ± *s*, beats/min)	68.78 ± 10.24	70.02 ± 10.96	0.523	.603
SBP (*x̄* ± *s*, mm Hg)	128.45 ± 15.76	129.90 ± 16.30	0.404	.687
DBP (*x̄* ± *s*, mm Hg)	73.80 ± 9.36	75.14 ± 10.25	0.611	.543
FBG (*x̄* ± *s*, mmol/L)	5.78 ± 1.20	6.10 ± 1.38	1.107	.272
TC (*x̄* ± *s*, mmol/L)	4.22 ± 1.10	4.26 ± 1.14	0.16	.874
TG (*x̄* ± *s*, mmol/L)	1.62 ± 0.40	1.72 ± 0.45	1.05	.297
HDL-C (*x̄* ± *s*, mmol/L)	1.15 ± 0.25	1.11 ± 0.37	0.567	.573
LDL-C (*x̄* ± *s*, mmol/L)	2.32 ± 0.70	2.38 ± 0.60	0.412	.682
LVEF (%)	69.40 ± 8.25	61.38 ± 9.12	4.125	<.001
NYHA classification (n [%])			11.35	.01
Class I	19 (47.50)	10 (25.00)		
Class II	17 (42.50)	14 (35.00)		
Class III	2 (5.00)	3 (7.50)		
Class IV	2 (5.00)	13 (32.50)		
Cr (*x̄* ± *s*, μmol/L)	78.80 ± 9.54	85.92 ± 9.80	3.293	.001
UA (*x̄* ± *s*, μmol/L)	319.42 ± 48.55	326.54 ± 56.78	0.603	.548

ACEI/ARB = angiotensin-converting enzyme inhibitors/angiotensin receptor blockers, Cr = creatinine, DBP = diastolic blood pressure, FBG = fasting blood glucose, HDL-C = high-density lipoprotein cholesterol, HR = heart rate, LDL-C = low-density lipoprotein cholesterol, LVEF = left ventricular ejection fraction, NYHA = New York Heart Association, PCI = percutaneous coronary intervention, SBP = systolic blood pressure, TC = total cholesterol, TG = triglycerides, UA = uric acid.

### 3.3. Logistic regression analysis of factors influencing NT-proBNP expression levels in UAP patients

Variables with statistically significant differences were included in the logistic regression model. The results showed that age, history of myocardial infarction, LVEF, NYHA classification, and serum creatinine (SCr) level were independent influencing factors of NT-proBNP expression levels in UAP patients. Among them, age, history of myocardial infarction, NYHA classification, and Cr level were risk factors, while LVEF was a protective factor. The odds ratio values were 1.175 (95% confidence interval [CI]: 1.096–1.248), 1.244 (95% CI: 1.185–1.460), 0.085 (95% CI: 0.074–0.096), 13.183 (95% CI: 2.824–61.532), and 1.118 (95% CI: 1.015–1.183), respectively (see Table 3 for details).

**Table 3:** Logistic regression analysis of factors influencing NT-proBNP expression levels in UAP patients.

Factor	Assignment	β	SE	Wald χ²	*P*	OR	95% CI for OR
Age	Actual value	0.133	0.052	6.559	.01	1.175	1.096–1.248
History of myocardial infarction	0 = no, 1 = yes	2.816	1.07	6.929	.008	1.244	1.185–1.460
LVEF	Actual value	−0.115	0.04	8.292	.004	0.085	0.074–0.096
NYHA classification	0 = class I or II, 1 = class III or IV	2.579	0.786	10.763	.001	13.183	2.824–61.532
Cr	Actual value	0.087	0.035	6.017	.014	1.118	1.015–1.183

CI = confidence interval, Cr = creatinine, LVEF = left ventricular ejection fraction, NT-proBNP = N-terminal pro-B-type natriuretic peptide, NYHA = New York Heart Association, OR = odds ratio, UAP = unstable angina pectoris.

### 3.4. Construction of the risk prediction model

**Figure 1.**
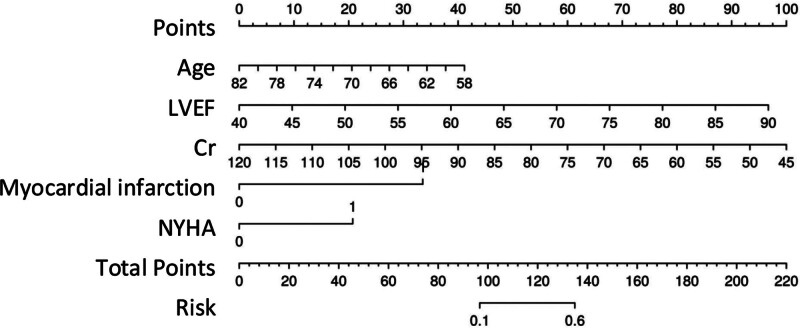
Nomogram model for predicting NT-proBNP expression levels in patients with UAP. Cr = creatinine, LVEF = left ventricular ejection fraction, NT-proBNP = N-terminal pro-B-type natriuretic peptide, NYHA = New York Heart Association, UAP = unstable angina pectoris.

Based on the results of the logistic regression analysis, a risk prediction model was developed, and a nomogram was constructed. The nomogram indicated that age, history of myocardial infarction, LVEF, NYHA classification, and Cr level had varying degrees of predictive value for NT-proBNP expression levels in UAP patients (see Fig. 1 for details).

### 3.5. Evaluation of the performance of the risk prediction model

**Figure 2.**
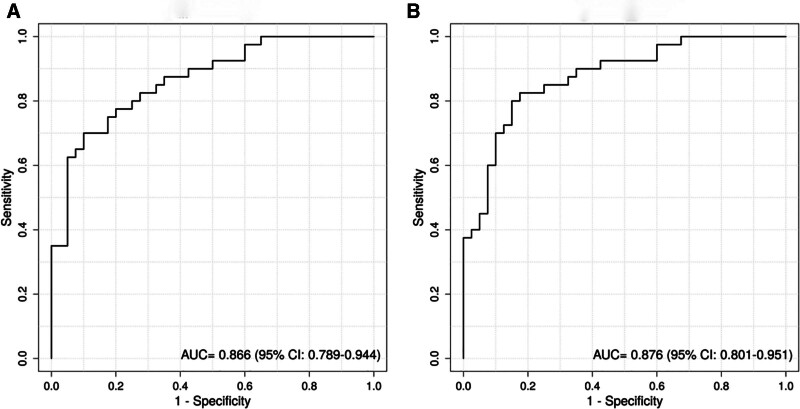
ROC curves of the risk prediction model. (A) ROC curve of the risk prediction model in the training set. (B) ROC curve of the risk prediction model in the validation set. AUC = area under the curve, CI = confidence interval, ROC = receiver operating characteristic.

The nomogram was constructed based on the risk prediction model, and the validation set was used to assess the accuracy of the model’s predictive performance. Receiver operating characteristic curves were plotted, and the area under the curve values for the training set and validation set were 0.866 (0.789–0.944) and 0.876 (0.801–0.951), respectively. The calibration curves showed good agreement with the ideal reference line, and the decision curve analysis demonstrated higher net clinical benefit compared to the 2 extreme curves. These results indicate that the model has good discrimination, calibration, and net clinical benefit (see Fig. 2 for details).

### 3.6. Calibration curve and decision curve of the risk prediction model

**Figure 3.**
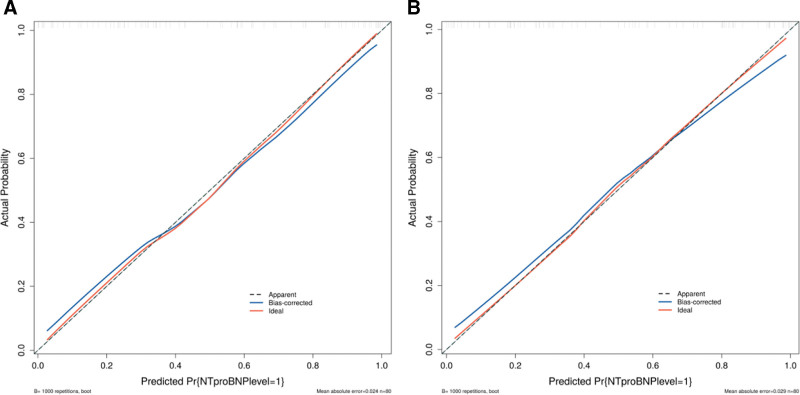
Calibration curves of the risk prediction model. (A) Calibration curve of the risk prediction model in the training set. (B) Calibration curve of the risk prediction model in the validation set. NT-proBNP = N-terminal pro-B-type natriuretic peptide.

**Figure 4.**
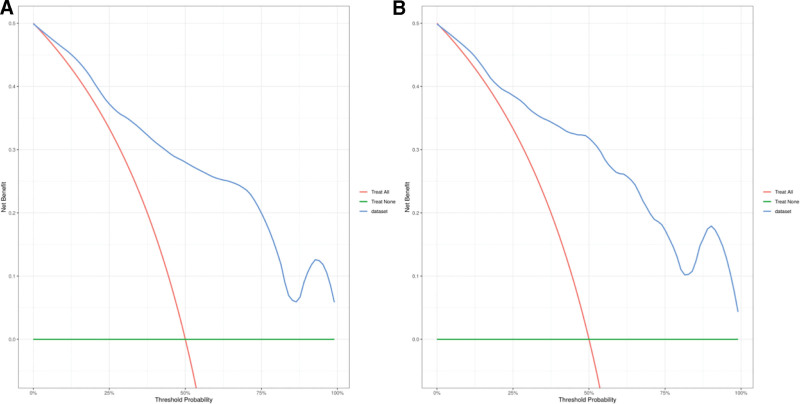
Decision curve analysis of the risk prediction model. (A) Decision curve of the risk prediction model in the training set. (B) Decision curve of the risk prediction model in the validation set.

The calibration curves of the risk prediction model in both the training and validation sets were close to the reference (ideal) line, indicating good agreement between the predicted risk and the actual risk of elevated NT-proBNP levels in UAP patients. Meanwhile, the decision curve analysis showed a higher net clinical benefit across a range of threshold probabilities, suggesting that the prediction model has good clinical applicability (see Figs. 3 and 4 for details).

## 4. Discussion

NT-proBNP is the stable N-terminal fragment of pro-B-type natriuretic peptide (BNP). Its level increases in response to ventricular wall stretching and elevated wall tension, making it a specific indicator in cardiac function assessment.^[12,13]^ Studies have also shown^[14]^ that hypoxia alone can stimulate the cardiac endocrine system, leading to increased NT-proBNP secretion. Therefore, NT-proBNP is widely used in risk stratification of patients with heart failure or coronary artery disease and is considered to be more sensitive than BNP in the evaluation of cardiac dysfunction.^[13]^

UAP results from coronary artery stenosis caused by atherosclerotic plaque deposition. Most patients present with evident chest tightness and chest pain, and many require coronary intervention to relieve symptoms.^[15]^ Although NT-proBNP expression in UAP patients is not as high as in acute myocardial infarction patients, it still shows an increasing trend and is higher than that in patients with stable angina.^[16,17]^

### 4.1. Influencing factors of NT-proBNP levels in UAP patients

This study showed that age, history of myocardial infarction, LVEF, NYHA classification, and SCr levels were significantly different between patients with low and high NT-proBNP levels (*P* < .05). Logistic regression analysis further confirmed that these 5 factors were independent predictors of NT-proBNP expression in UAP patients.

Zhen et al^[18]^ reported that age is a major factor influencing NT-proBNP levels, which is consistent with our findings. With increasing age, left ventricular diastolic function gradually declines, affecting myocardial synthesis and secretion processes and thereby elevating NT-proBNP levels.^[19]^

In patients with a history of myocardial infarction, varying degrees of myocardial necrosis lead to impaired systolic and diastolic function, increased ventricular pressure, and consequently enhanced BNP secretion, resulting in elevated NT-proBNP levels.^[20]^

LVEF and NYHA classification are commonly used indicators for evaluating cardiac function. Lower LVEF and higher NYHA class indicate worse cardiac function in UAP patients. When LVEF improves, cardiac pumping ability is enhanced, left ventricular pressure decreases, and NT-proBNP synthesis by ventricular myocytes is reduced, subsequently lowering serum NT-proBNP levels.^[21]^ Jin et al^[10]^ also identified LVEF as a protective factor for NT-proBNP levels in patients with heart disease, which aligns with this study.

SCr reflects renal function status. When renal function is impaired, creatinine excretion decreases, leading to elevated SCr levels. Increased Cr further exacerbates cardiac dysfunction and stimulates ventricular myocytes to secrete higher amounts of NT-proBNP.^[22]^ Chen et al^[23]^ also demonstrated that Cr level affects NT-proBNP expression, supporting the conclusions of this study.

In addition to renal dysfunction affecting NT-proBNP clearance, elevated NT-proBNP may also reflect underlying hemodynamic disturbances that contribute to renal hypoperfusion and further deterioration of renal function. This bidirectional interaction between cardiac and renal dysfunction is consistent with the concept of cardiorenal syndrome and should be considered when interpreting NT-proBNP levels. Although NT-proBNP has established age- and sex-specific reference ranges in clinical practice, median-based grouping was used in this study for statistical modeling purposes. This approach may limit direct clinical interpretability, and future studies should explore clinically validated cutoff values.

### 4.2. Intervention strategies

Based on the above factors, the following intervention strategies are proposed: For UAP patients of advanced age, especially elderly patients, clinical monitoring should be strengthened to promptly identify and manage various factors that may lead to deterioration of cardiac function, such as arrhythmias and myocardial ischemia. History of myocardial infarction: For UAP patients with a history of myocardial infarction, active cardiac rehabilitation and secondary prevention should be implemented, mainly including control of blood pressure, blood lipids, and blood glucose, initiation of appropriate pharmacological therapy to improve myocardial remodeling, and regular evaluation of cardiac function. For patients with low LVEF, measures to enhance myocardial contractility should be considered, and cardiac preload and afterload should be optimized to improve cardiac function. According to the NYHA classification of UAP patients, individualized treatment plans should be developed, and specific exercise-based rehabilitation strategies should be determined. For patients with poor cardiac function, stricter fluid management, cardiac monitoring, and pharmacological treatment should be performed. For UAP patients with abnormal Cr levels, renal insufficiency may be present; therefore, NT-proBNP levels should be carefully assessed, renal diseases should be treated actively, and renal function protection should be strengthened to minimize the impact on NT-proBNP expression in UAP patients. Through the above interventions, cardiac function may be optimized, and serum NT-proBNP levels may be regulated.

In this study, a logistic regression-based risk prediction model was constructed and internally validated using the validation dataset. The area under the curve values of the model in the training and validation sets were 0.866 (0.789–0.944) and 0.876 (0.801–0.951), respectively. The calibration curves in both the training and validation sets were close to the reference line, and the decision curves were higher than the 2 extreme curves, indicating that the model exhibits good discrimination, calibration, and net clinical benefit. This suggests that the model has certain predictive value for NT-proBNP expression levels in UAP patients and can provide substantial clinical support. However, this study still has some limitations. As a retrospective study, potential confounding factors and information bias cannot be completely excluded, although we attempted to collect complete data and ensure comparability between the 2 groups. In addition, this is a single-center study with a relatively small sample size, which may limit the generalizability of the results. The influencing factors included in the analysis were relatively limited, and factors such as atrial fibrillation and coronary stenosis were not included, although other relevant variables were analyzed as comprehensively as possible. The study duration was short, and long-term follow-up data were lacking. In future research, more refined designs with large sample sizes, long-term follow-up, and multicenter collaboration are needed to compensate for these limitations.

It should be emphasized that this study is a retrospective observational analysis; therefore, the associations identified between NT-proBNP levels and clinical variables reflect correlations rather than causal relationships. Causal inference cannot be established, and the findings should be interpreted with caution.

In conclusion, age, history of myocardial infarction, LVEF, NYHA classification, and Cr level are influencing factors of NT-proBNP levels in UAP patients. A prediction model constructed based on these indicators can provide a reference for clinically screening UAP patients with high NT-proBNP levels. Clinicians should pay close attention to elderly patients, those with a history of myocardial infarction, higher NYHA classification, and elevated Cr levels, and actively adopt effective treatment strategies to increase LVEF, improve cardiac function, and thereby control NT-proBNP expression levels.

## Author contributions

**Conceptualization:** Zheng Sun.

**Data curation:** Zheng Sun.

**Formal analysis:** Zheng Sun.

**Funding acquisition:** Zheng Sun.

**Investigation:** Zheng Sun.

**Writing – original draft:** Zheng Sun.

**Writing – review & editing:** Zheng Sun.
